# A Compend of Human Anatomy, Including the Anatomy of the Viscera

**Published:** 1891-04

**Authors:** 


ESSENTIALS OF MINOR SURGERY AND BANDAGING, WITH AN APPENDIX ON VENEREAL DISEASES. Arranged in the form of questions and answers. Prepared especially for students of medicine. By EDWARD MARTIN, A. M., M. D., Instructor in Operative Surgery, University of Pennsylvania; Surgeon of the Howard Hospital; Assistant Surgeon to the University, etc. Illustrated. Saunders' Question Compends, No. 12. Philadelphia: W. B. Saunders, 913 Walnut street. 1890.

This little book covers an extensive field of surgery, comprising bandaging, the preparation of antiseptic materials for use during and after operations, anesthetics, venereal diseases, etc. The descriptions are concise, yet clear, and evidently written by one who is perfectly familiar with the subjects considered. J. P.

